# YaHS: yet another Hi-C scaffolding tool

**DOI:** 10.1093/bioinformatics/btac808

**Published:** 2022-12-16

**Authors:** Chenxi Zhou, Shane A McCarthy, Richard Durbin

**Affiliations:** Department of Genetics, University of Cambridge, Cambridge CB2 3EH, UK; Wellcome Sanger Institute, Wellcome Genome Campus, Cambridge CB10 1SA, UK; Department of Genetics, University of Cambridge, Cambridge CB2 3EH, UK; Wellcome Sanger Institute, Wellcome Genome Campus, Cambridge CB10 1SA, UK; Department of Genetics, University of Cambridge, Cambridge CB2 3EH, UK; Wellcome Sanger Institute, Wellcome Genome Campus, Cambridge CB10 1SA, UK

## Abstract

**Summary:**

We present YaHS, a user-friendly command-line tool for the construction of chromosome-scale scaffolds from Hi-C data. It can be run with a single-line command, requires minimal input from users (an assembly file and an alignment file) which is compatible with similar tools and provides assembly results in multiple formats, thereby enabling rapid, robust and scalable construction of high-quality genome assemblies with high accuracy and contiguity.

**Availability and implementation:**

YaHS is implemented in C and licensed under the MIT License. The source code, documentation and tutorial are available at https://github.com/sanger-tol/yahs.

**Supplementary information:**

Supplementary data are available at *Bioinformatics* online.

## 1 Introduction

The rapid revolution of long-read, single-molecule DNA sequencing technologies in read length, base accuracy and per-base cost is driving a golden age for *de novo* genome assembly. Multiple genome sequencing projects have been launched in the past few years, such as the Earth Biogenome Project (Lewin *et al.*, 2018), the Vertebrate Genomes Project (Rhie *et al.*, 2021) and the Darwin Tree of Life Project (DToL, Blaxter *et al.*, 2022) aiming to assemble high quality, chromosome-scale genomes for many thousands of species across a range of genome sizes, complexity and ploidy. Despite the technological advances, the assembly of reference-quality genomes with long-read sequencing data alone remains elusive (Amarasinghe *et al.*, 2020). Long-distance linkage information, such as physical maps, genetic maps, optical maps and Hi-C contact maps, is often used to construct chromosome-scale scaffolds from contigs. Hi-C is a sequencing-based proximity ligation assay that provides contact information between pairs of loci, originally designed to study the 3D structure of the genome inside a cell nucleus (Lieberman-Aiden *et al.*, 2009). Since the contact frequency between loci pairs strongly correlates with separation on the genome, Hi-C has rapidly gained popularity as an economical method for generating chromosome-scale scaffolds (Burton *et al.*, 2013; Dudchenko *et al.*, 2017). Several scaffolding tools have been developed for the construction of chromosome-scale assembly with Hi-C data including LACHESIS (Burton *et al.*, 2013), HiRise (Putnam *et al.*, 2016), 3D-DNA (Dudchenko *et al.*, 2017), ALLHiC (Zhang *et al.*, 2019), SALSA2 (Ghurye *et al.*, 2019) and pin_hic (Guan *et al.*, 2021). Each of these has its own limitations, and the results are affected by various factors such as genome complexity and repeat content, Hi-C library preparation and sequencing coverage (Ghurye *et al.*, 2019; Guan *et al.*, 2021; Kadota *et al.*, 2020).

In this article, we introduce YaHS, another scaffolding tool which constructs chromosome-scale scaffolds utilizing Hi-C data. YaHS follows a standard framework of Hi-C scaffolding pipelines: map Hi-C reads to input contigs, break contigs where necessary to correct assembly errors, build a contact matrix, construct and prune a scaffolding graph and finally output scaffolds. The tool assumes the input contigs are derived from a single haplotype. If the genome assembly contains haplotypic duplications, they should be removed first. The core idea behind YaHS, which distinguishes it from other Hi-C scaffolding tools, is a novel method for building the contact matrix (Supplementary Methods). This method enables more accurate inferences of contig joins. In comparisons with SALSA2 and pin_hic, two recently published Hi-C scaffolding tools, applied to both simulated and real data, YaHS generated genome assemblies of higher accuracy and contiguity, and was more robust to assembly errors.

## 2 Results

### 2.1 Overview

The scaffolding process starts with mapping Hi-C reads to the input contigs, which falls outside of the scope of YaHS. The Arima Genomics mapping pipeline was employed in this study which consists of four major steps: read mapping in single-end read mode, read filtering for chimeric joins across ligation junctions, read pairing and PCR duplicate removal (https://github.com/ArimaGenomics/mapping_pipeline). YaHS takes the alignment file (either in BED format or BAM format) to first optionally break contigs at positions lacking Hi-C coverage which are potential assembly errors. Scaffolding then proceeds in multiple rounds. In each round YaHS builds a contact matrix by splitting each contig into chunks of a certain size (i.e. resolution) and assigns Hi-C contact signals into cells of chunk pairs. Here, we refer to cells within contigs as *intra*-cells and between contigs as *inter*-cells. The Hi-C contact frequencies are counted for each cell. To calculate the joining score of a pair of contigs, the contact frequencies of the *inter*-cells between them are normalized by expected values which are estimated by the medians of the *intra*-cells at the same separations and then used to calculate a weighted sum. The fundamental idea is that the *inter*-cells of neighbouring contigs on a scaffold should have similar contact frequencies to *intra*-cells at the same separations. The joining scores of a contig pair are calculated in all four possible orientations and the one with the largest score is selected as the joining orientation. YaHS also optionally takes account of the restriction enzymes used in the Hi-C library, and if so, the cell contact frequencies are normalized first by the corresponding number of cutting sites. YaHS next builds a scaffolding graph with contigs as nodes and contig joins as edges which are weighted by the joining scores calculated in the previous step. The graph is simplified by a series of operations including filtering low-score edges, trimming tips, trimming blunt ends, solving repeats, removing transitive edges, removing bubbles, resolving ambiguous orientations, trimming weak edges and removing ambiguous edges. Finally, the graph is traversed to assemble scaffolds along contiguous paths. A second step of assembly error correction is optionally performed to break scaffolds at positions of contig joins without sufficient Hi-C coverage. YaHS runs a hierarchical joining process with multiple rounds of scaffolding at decreasing resolutions (increasing chunk sizes). In each round, the scaffolds generated in the previous round are used as the input except for the first round when the contigs are used. See Supplementary Methods for further details.

### 2.2 On simulated human genome assemblies

**Fig. 1.**
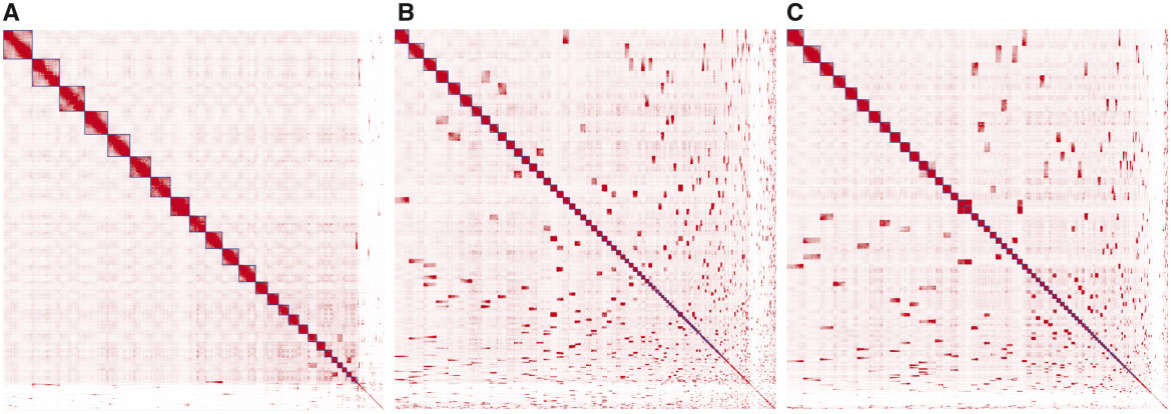
Hi-C contact maps of genome assemblies constructed with YaHS (**A**), SALSA2 (**B**) and pin_hic (**C**) for the simulated T2T data without contig errors. The intensity of colour indicates the density of Hi-C read pairs shared between the positions on the *x*- and *y*-axis, with darker pixels indicating higher densities. The blocks highlighted with squares along the main diagonal are scaffolds constructed by the tools. The dark off-diagonal blocks indicate scaffold pairs that could be further joined for construction of larger scaffolds. The contact maps were plotted with Juicebox (Durand *et al.*, 2016)

We randomly split the Telomere-to-Telomere (T2T) human genome assembly (T2T-CHM13, Nurk *et al.*, 2022) into 100 kb to 1 Mb chunks and ended up with a simulated assembly of 5483 contigs with an N50 of 715 kb. The T2T Arima Hi-C data were downloaded from NCBI (accession SRX10230901–SRX10230903) and mapped to the simulated assembly for the reconstruction of the genome with YaHS, SALSA2 and pin_hic. YaHS assembled over 92% sequences into 25 major scaffolds (>35 Mb). The N50 and N90 were 132.6 Mb (L50 = 9) and 36.5 Mb (L90 = 23), respectively (Fig. 1A). In contrast, the N50 and N90 of SALSA2 assembly were 43.9 Mb (L50 = 22) and 3.3 Mb (L90 = 103), respectively (Fig. 1B), and the N50 and N90 of pin_hic assembly were 51.5 Mb (L50 = 19) and 8.5 Mb (L90 = 68), respectively (Fig. 1C). We used QUAST-LG (Mikheenko *et al.*, 2018) to map the scaffolds to the T2T-CHM13 genome assembly for quality assessment. Three types of misassemblies were considered: relocations (gaps on scaffolds), inversions (misorientated contigs) and translocations (misjoins of inter-chromosomal contigs). The numbers of relocations and inversions reported by QUAST-LG for YaHS, SALSA2 and pin_hic assemblies were 40 and 21, 262 and 55, and 171 and 115, respectively. No translocation was reported for any of the three assemblies.

To evaluate the performance of these tools on assemblies with errors, we generated another assembly by randomly introducing 25 erroneous contigs into the previous one. These included 10 with a single misjoin of two *intra*-chromosomal contigs, 10 with a single misjoin of two *inter*-chromosomal contigs and 5 with two misjoins of any three contigs—a total of 30 assembly errors. The joining orientations were randomly determined. In total, these affected 32.1 Mb of contig sequences. The new assembly comprised 5453 contigs with an N50 of 718 kb. YaHS corrected 28 errors out of the 30. The two errors missed were on a double-misjoined contig with two closely located contigs from chr10 (a gap of 1.2 Mb) flanking a short contig (170 kb) from chr19 (Supplementary Fig. S1). The error detection in this scenario was more challenging. SALSA2 corrected 14 errors out of the 30. No false positive contig breaks were made by YaHS and SALSA2. Pin_hic did not do assembly error correction explicitly but instead broke scaffolds at suspicious misassembled positions at the end of the scaffolding process. The contiguity of the scaffolding resulting from each the three tools was similar to that of the previous error-free assembly: all statistics for YaHS remained identical; SALSA2 had a slightly larger N50 of 46.1 Mb and smaller L50 of 20; pin_hic ended up with a more fragmented assembly with N50 and N90, respectively decreasing to 37.5 Mb (L50 = 28) and 5.8 Mb (L90 = 98). The scaffolding errors for YaHS reported by QUAST-LG also remained similar with two more relocations and two extra translocations due to the uncorrected contig errors. In contrast, significantly more errors were reported for the other two assemblies. The numbers of relocations, inversions and translocations were 278, 18 and 63, respectively for the SALSA2 assembly, and 188, 22 and 123, respectively for the pin_hic assembly.

### 2.3 On a real human genome assembly

We constructed scaffolds from a real PacBio CHM13 contig level genome assembly (NCBI accession GCA_000983455.2) with the three tools. The genome assembly consists of 4961 contigs, with a total size of 2.94 Gb, an N50 of 10.5 Mb (L50 = 82) and an N90 of 972 kb (L90 = 403). The same Hi-C data as described in the previous section were used. YaHS and SALSA2 made 125 and 20 contig breaks, respectively. The N50 and N90 statistics of the genome assemblies generated by YaHS, SALSA2 and pin_hic were 147.7 Mb (L50 = 8) and 39.4 Mb (L90 = 22), 102.7 Mb (L50 = 11) and 8.1 Mb (L90 = 42), and 87.9 Mb (L50 = 12) and 14.6 Mb (L90 = 38), respectively. When compared them to the T2T-CHM13 genome assembly, the total numbers of misassemblies reported by QUAST-LG were 2627, 2835 and 2799, respectively. Most of them were contig misassemblies generated during the PacBio sequence assembly process, constituting 2127, 2260 and 2258 misassemblies, respectively. The YaHS result represented fewer contig misassemblies since it made more contig error corrections. For scaffold misassemblies, the numbers of relocations, inversions and translocations reported by QUAST-LG were 442, 12 and 46, respectively for the YaHS assembly, 468, 16 and 91, respectively for the SALSA2 assembly and 446, 6 and 89, respectively for the pin_hic assembly. The Hi-C contact maps of these genome assemblies are showed in Supplementary Figure S2.

### 2.4 On Darwin Tree of Life assemblies

We applied the three tools to the construction of genome assemblies for 15 DToL species across a range of taxonomic groups, genome sizes and initial assembly quality. YaHS consistently generated assemblies with higher contiguity compared to SALSA2 and pin_hic particularly for the L90 statistics (Supplementary Table S1, Supplementary Figures S3–S17).

In particular, we constructed a genome assembly for the oak bush cricket (*Meconema thalassinum*, ToLID: iqMecThal1) which has a very large genome size estimated to be over 9 Gb. The initial genome assembly consisted of 2093 contigs of 9054 Mb with an N50 of 10.7 Mb (L50 = 229). YaHS detected 268 assembly errors. In the final assembly, three scaffolds longer than 1349 Mb constituted over 50% of the assembly, and 13 scaffolds longer than 179.6 Mb constituted 90% of the assembly. The largest scaffold was 2087 Mb. In comparison, the N50 and N90 for the SALSA2 assembly were 79.0 Mb (L50 = 18) and 3.5 Mb (L90 = 311), respectively, and for the pin_hic assembly were 202.4 Mb (L50 = 12) and 14.2 Mb (L90 = 82), respectively. See Supplementary Figure S3 for the Hi-C contact maps of these genome assemblies. For the fungus chicken of the woods (*Laetiporus sulphureus*, ToLID: gfLaeSulp1), YaHS and pin_hic made several incorrect joins at the telomere ends (Supplementary Fig. S17).

## 3 Conclusion

YaHS is a fast, reliable and accurate tool for the construction of chromosome-scale scaffolds with Hi-C data that is now being used routinely by the DToL project and others. It consistently outperforms other state-of-the-art Hi-C scaffolding tools in both genome assembly accuracy and contiguity across a wide range of species and genome sizes, and initial assembly quality. It learns its parameters from the data so is robust to Hi-C data with different genomic separation distributions, including generated with different protocols. It is open source, easy to use and well documented.

## Supplementary Material

btac808_Supplementary_Data

## Data Availability

No new data were generated in support of this research. All data analysed are available from INSDC, with accessions given in the main text or Supplementary Table S1.
